# Intratumor Heterogeneity of HLA-G Expression in Cancer Lesions

**DOI:** 10.3389/fimmu.2020.565759

**Published:** 2020-11-19

**Authors:** Xia Zhang, Aifen Lin, Qiu-Yue Han, Jian-Gang Zhang, Qiong-Yuan Chen, Yao-Han Ye, Wen-Jun Zhou, Hui-Hui Xu, Jun Gan, Wei-Hua Yan

**Affiliations:** ^1^ Biological Resource Center, TaiZhou Hospital of Zhejiang Province, Taizhou Enze Medical Center (Group), LinHai, China; ^2^ Medical Research Center, TaiZhou Hospital of Zhejiang Province, Taizhou Enze Medical Center (Group), LinHai, China

**Keywords:** HLA-G, tumor, heterogeneity, isoform, antibody, colorectal cancer, esophageal cancer

## Abstract

Signaling pathway between human leukocyte antigen (HLA)-G and immune inhibitory receptors immunoglobulin-like transcript (ILT)-2/4 has been acknowledged as one of immune checkpoints, and as a potential target for cancer immunotherapy. Like other immune checkpoints, inter- and even intratumor heterogeneity of HLA-G could render a rather complexity for HLA-G-target immunotherapy. However, little information for intratumor heterogeneity of HLA-G is available. In this study, HLA-G expression in a serial section of colorectal cancer (CRC) lesions from three CRC patients (each sample with serial section of 50 slides, 10 randomized slides for each antibody), three different locations within a same sample (five CRC), and three case-matched blocks that each includes 36 esophageal cancer samples, were evaluated with immunohistochemistry using anti-HLA-G antibodies (mAbs 4H84, MEM-G/1 and MEM-G/2 probing for all denatured HLA-G isoforms, 5A6G7, and 2A12 probing for denatured HLA-G5 and HLA-G6 isoforms). Our results revealed that, in addition to the frequently observed inter-tumor heterogeneity, intratumor heterogeneous expression of HLA-G is common in different areas within a tumor in CRC and esophageal cancer samples included in this study. Moreover, percentage of HLA-G expression probed with different anti-HLA-G antibodies also varies dramatically within a tumor. Given HLA-G has been considered as an important immune checkpoint, intratumor heterogeneity of HLA-G expression, and different specificity of anti-HLA-G antibodies being used among studies, interpretation and clinical significance of HLA-G expression in cancers should be with caution.

## Introduction

Immune suppressive functions induced by the interaction between human leukocyte antigen-G (HLA-G) and its immune inhibitory receptors, the immunoglobulin-like transcripts (ILTs), have been widely acknowledged (1). Receptors ILT-2 and ILT-4 express on various immune cells, the immune tolerogenic effects induced by HLA-G are comprehensive (2). Due to alternative splicing of its primary transcripts, seven confirmed HLA-G isoforms (HLA-G1~HLA-G7), and recently predicted novel isoforms such as lacking a transmembrane region and α1 domain have been reported (3).

In the context of cancers, different degree of inter-tumor HLA-G expression has been observed in most histological types of cancers studied, and the significance of HLA-G/ILTs signaling pathway as an immune checkpoint in cancer biology has been highlighted (4). Look back to its expression firstly observed in cancer, the melanoma lesions in 1998 (5), immune tolerance induced by HLA-G has been solidified by large numbers of studies both *in vitro* and *in vivo* preclinical experimental animal models (6–8).

HLA-G/ILTs binding can inhibit the proliferation of natural killer cells (NK), T and B lymphocytes and maturation and antigen presentation of dendritic cells (DC), suppress NK and T cell’s cytotoxic function, B cell’s immunoglobulin production and neutrophils’ reactive oxygen species production and phagocytosis capability (9–11). To the contrary, HLA-G/ILTs binding can promote myeloid-derived suppressor cells (MDSC) proliferation and polarize M1 macrophages towards to M2 type (12, 13). Moreover, immune tolerance can be induced by HLA-G-bearing exosomes between cells at long-distance, and by cellular membrane fragments containing HLA-G through trogocytosis in a close cell-to-cell contact manner (14, 15). In preclinical murine models, HLA-G could promote tumor immune escape and growth through murine MDSC proliferation and Th2 cytokine production, or reduce T and B cell tumor infiltrate, impair B cell immune responses in immunocompetent mice (8, 16). Findings also revealed that HLA-G expression in ovarian cancer cells could enhance the tumor cell migration and metastasis in tumor-bearing immunodeficient nude mice through induction of matrix metalloproteinase-15 (MMP-15) expression (7, 17). Moreover, a recent study showed that depletion of CD4^low^HLA-G^+^ T cells could favor the castration-resistance prostate cancer therapy (18). Echoing the above mentioned *in vitro* and *in vivo* preclinical experimental observations, lesion HLA-G expression was observed to be closely associated with tumor metastasis, poor tumor cell differentiation, advanced disease stage and worse survival in a variety of cancers in clinical settings (14).

Inter- and intratumor heterogeneity of immune checkpoints is the main obstacle for immune checkpoint inhibitor (ICI) immunotherapy. Consequently, the benefits of the ICI therapy varies dramatically among patients (19). As a new immune checkpoint, the inter-tumor heterogeneous pattern of HLA-G expression is well evidenced; however, information for the intratumor heterogeneity of HLA-G is very limited. Previous studies revealed that the degree of HLA-G or its receptors ILT2/4 expression varies markedly among different locations in a primary renal cell cancer tumor lesion, indicating the complexity of intratumor heterogeneity of HLA-G and its receptor expression (3, 20).

In this study, inter- and intratumor heterogeneity of HLA-G expression was evaluated with immunohistochemistry using a panel of anti-HLA-G antibodies (mAbs 4H84, MEM-G/1 and MEM-G/2 probing for all denatured HLA-G isoforms, 5A6G7 and 2A12 probing for denatured HLA-G5 and HLA-G6 isoforms) in a serial section of colorectal cancer lesions from three CRC patients, three different locations within a same sample from five CRC patients, and three case-match blocks that each includes 36 esophageal cancer samples, and our findings solidify the heterogeneity of HLA-G in cancers.

## Materials and Methods

### Tumor Lesion Specimen

Tumor lesion specimen and clinical records were retrospectively reviewed. In this study, three CRC lesions #598937 (Female, 65 years, AJCC stage IIIA), #624267 (Female, 72 years, AJCC stage I A) and #681878 (Female, 80 years, AJCC stage I A; **Table 1**), and each sample was serially sectioned for 50 slides. Slides from three different locations within a same sample from another five CRC samples were obtained #1022488 (Male, 49 years, AJCC stage III B), #1022363 (Male, 70 years, AJCC stage I A), #1020932 (Male, 75 years, AJCC stage IV A), #1023081 (Male, 75 years, AJCC stage II A) and #444345 (Male, 86 years, AJCC stage II A; **Table 2**). Furthermore, slides from three case-matched blocks that each includes 36 esophageal squamous cell carcinoma (ESCC) samples were included in the study. Among 36 ESCC patients (27 male and nine female; median age: 58 years; range from 47 to 79 years), there were one patient with stage I B, six patients with II A, 14 patients with II B, seven patients with III A, seven patients with III B, and one patient with III C. The detailed clinical information was shown in **Table 3**. The clinicopathological findings were determined according to 7th American Joint Committee on Cancer (AJCC) Tumor-Node-Metastasis (TNM) staging system (21). None of them received radiotherapy, chemotherapy, or other medical interventions before the study. All these patients were diagnosed and treated at Taizhou Hospital of Zhejiang Province, China, and samples were retrieved by Biological Resource Center, Taizhou Hospital of Zhejiang Province (National Human Genetic Resources Platform of China YCZYPT [2017]02). Written informed consent was obtained from each participant before the surgical operation, and this study was approved by Medical Ethics Review Board of Taizhou Hospital of Zhejiang Province.

**Table 1:** Percentage of HLA-G expression in serial section of colorectal cancer lesions.

Samples		Antibodies	Percentage of HLA-G positive tumor cells (%)											*p*
			^#^1	^#^2	^#^3	^#^4	^#^5	^#^6	^#^7	^#^8	^#^9	^#^10	Mean	
CRC ^#^598937														
Female, 65 years, AJCC stage IIIA														
Group 1 (All isoforms)		mAb 4H84	88.8	86.9	81.9	88.1	85.0	86.3	91.9	91.3	85.6	91.3	87.71	<0.001
		mAb MEM-G/1	65.0	55.0	65.0	61.3	62.9	62.5	67.5	72.5	76.3	62.5	65.05	
		mAb MEM-G/2	57.5	65.0	68.8	57.5	77.5	77.5	70.0	67.5	75.0	77.5	69.38	
Group 2 (HLA-G5/6)		mAb 5A6G7	55.0	45.0	60.0	45.0	41.3	53.8	58.3	50.0	60.0	76.3	54.47	0.108
		mAb 2A12	47.5	48.8	50.0	41.3	53.8	46.3	43.8	50.0	57.5	35.0	47.40	
CRC ^#^624267														
Female, 72 years, AJCC stage IA														
Group 1 (All isoforms)		mAb 4H84	94.5	94.1	95.0	94.1	94.1	92.7	94.1	94.1	94.5	92.3	93.95	0.453
		mAb MEM-G/1	94.5	89.1	94.1	93.6	94.5	94.1	94.5	93.6	91.8	94.1	93.39	
		mAb MEM-G/2	93.6	92.7	93.2	94.1	95.0	93.6	93.6	92.7	92.7	94.5	93.57	
Group 2 (HLA-G5/6)		mAb 5A6G7	92.7	90.2	90.9	86.8	78.2	92.7	90.0	88.6	93.2	88.6	89.19	0.190
		mAb 2A12	91.1	93.2	80.0	89.8	89.9	85.9	89.5	82.3	77.3	84.1	86.31	
CRC ^#^681878														
Female, 80 years, AJCC stage IA														
Group 1 (All isoforms)		mAb 4H84	81.9	80.0	80.6	82.5	84.4	80.6	80.6	84.4	85.0	91.3	83.13	<0.001
		mAb MEM-G/1	43.8	45.0	27.5	46.3	50.1	32.5	43.8	60.0	26.3	33.8	40.91	
		mAb MEM-G/2	0.00	36.3	21.3	18.8	14.4	22.5	6.30	5.00	10.6	26.3	16.15	
Group 2 (HLA-G5/6)		mAb 5A6G7	68.3	85.6	76.3	68.8	61.9	61.9	60.6	52.5	39.4	63.8	63.91	0.105
		mAb 2A12	69.4	62.5	61.3	68.1	70.6	71.9	72.5	83.8	76.9	72.5	70.95	

**Table 2:** Percentage of HLA-G expression in different zones of colorectal cancer lesions.

Sample	Sex	Age	AJCCStage	Percentage of HLA-G positive tumor cells (%)				
				4H84	MEM-G/1	MEM-G/2	5A6G7	2A12
CRC ^#^1022488	Male	49	III B					
Zone 1				0.57	2.43	0.27	9.16	2.84
Zone 2				0.71	1.67	0.21	20.29	9.69
Zone 3				2.00	2.22	0.00	7.18	4.42
CRC ^#^1022363	Male	70	I A					
Zone 1				14.88	9.25	2.60	22.05	11.90
Zone 2				50.00	0.00	0.00	4.12	24.80
Zone 3				22.00	0.44	0.67	9.42	12.89
CRC ^#^1020932	Male	75	IV A					
Zone 1				45.00	8.93	8.74	32.61	24.53
Zone 2				15.58	26.38	7.13	16.13	19.50
Zone 3				13.19	7.00	0.50	24.19	18.13
CRC ^#^1023081	Male	75	II A					
Zone 1				59.49	34.14	56.57	4.33	16.43
Zone 2				15.19	55.20	59.23	32.3	19.70
Zone 3				36.32	24.48	14.10	2.74	7.58
CRC ^#^0444345	Male	86	II A					
Zone 1				45.30	25.40	34.80	16.23	0.00
Zone 2				13.00	58.82	32.35	41.14	30.59
Zone 3				32.74	33.23	37.42	31.42	27.10

**Table 3:** Percentage of HLA-G expression in different blocks of esophageal squamous cell carcinoma.

No.	Sex	Age	AJCC stage	Percentage of HLA-G positive tumor cells (%)					
				Blocks	4H84	MEM-G/1	MEM-G/2	5A6G7	2A12
1	Male	61	III B	1#	40	10	0	0	0
				2#	58	10	0	0	0
				3#	0	0	5	0	0
2	Male	62	II B	1#	30	30	10	0	0
				2#	65	60	40	0	0
				3#	80	75	45	0	0
3	Female	54	III A	1#	65	80	45	1	0
				2#	98	30	0	0	40
				3#	80	80	20	0	0
4	Female	47	II B	1#	60	10	40	0	0
				2#	80	30	0	0	0
				3#	98	90	90	80	85
5	Male	60	II A	1#	95	90	90	0	80
				2#	70	80	30	0	0
				3#	95	85	80	0	85
6	Male	53	II B	1#	80	90	70	3	20
				2#	80	60	3	0	0
				3#	55	70	5	0	3
7	Male	56	II B	1#	60	60	0	0	0
				2#	60	55	0	0	0
				3#	95	80	85	10	0
8	Female	72	II A	1#	80	85	65	0	0
				2#	95	85	15	20	45
				3#	90	90	80	5	1
9	Male	72	III A	1#	90	90	90	70	30
				2#	70	90	60	0	0
				3#	95	85	40	0	1
10	Male	65	I B	1#	0	0	0	0	0
				2#	0	0	0	0	0
				3#	40	10	0	0	0
11	Male	51	II B	1#	0	0	0	0	0
				2#	0	0	0	0	0
				3#	20	0	0	0	0
12	Male	56	II B	1#	0	0	0	0	0
				2#	30	20	0	0	0
				3#	75	20	0	0	0
13	Male	58	III A	1#	0	0	0	0	0
				2#	20	0	0	0	0
				3#	40	0	80	0	0
14	Male	59	II B	1#	70	55	55	0	0
				2#	40	10	0	0	0
				3#	98	5	70	0	0
15	Male	79	II A	1#	35	15	30	10	10
				2#	35	30	20	0	0
				3#	85	5	55	0	10
16	Male	57	II B	1#	70	5	80	60	10
				2#	30	65	40	0	0
				3#	98	60	30	0	0
17	Female	58	III A	1#	70	80	10	0	0
				2#	80	60	90	0	0
				3#	40	0	80	0	0
18	Male	59	III A	1#	95	90	80	0	60
				2#	95	95	90	2	40
				3#	95	85	70	65	45
19	Male	48	II A	1#	20	0	10	0	10
				2#	10	80	10	0	0
				3#	80	0	0	0	0
20	Male	59	III B	1#	80	80	0	0	0
				2#	60	0	0	0	0
				3#	40	2	0	0	0
21	Female	58	II B	1#	60	60	0	0	0
				2#	40	0	0	0	0
				3#	30	0	0	0	0
22	Female	73	II B	1#	60	0	0	0	0
				2#	65	0	0	0	0
				3#	70	0	0	0	0
23	Male	50	III A	1#	3	0	0	0	0
				2#	0	0	0	0	0
				3#	15	0	0	0	0
24	Female	58	III B	1#	80	60	30	0	0
				2#	85	0	20	0	0
				3#	85	70	60	0	0
25	Male	50	III B	1#	60	10	0	0	0
				2#	70	0	0	0	0
				3#	75	30	30	0	0
26	Female	50	III C	1#	80	55	0	0	0
				2#	70	0	55	0	0
				3#	85	80	0	0	0
27	Male	70	II B	1#	60	40	0	0	0
				2#	10	0	0	0	0
				3#	20	10	0	0	0
28	Male	49	II A	1#	85	80	80	80	0
				2#	70	0	80	0	3
				3#	95	90	85	80	70
29	Male	55	II B	1#	90	40	30	0	0
				2#	10	0	0	0	0
				3#	85	20	40	0	0
30	Male	53	II A	1#	95	80	80	0	2
				2#	60	2	15	2	2
				3#	90	70	80	30	10
31	Male	59	III B	1#	65	2	10	0	0
				2#	80	0	55	0	0
				3#	85	80	70	0	0
32	Male	59	III B	1#	90	90	90	60	0
				2#	85	0	40	0	0
				3#	95	90	90	30	0
33	Male	51	III A	1#	95	80	70	0	2
				2#	90	0	80	3	0
				3#	95	30	20	0	2
34	Female	53	II B	1#	90	3	80	0	0
				2#	80	0	0	0	0
				3#	90	85	60	0	0
35	Male	69	II B	1#	0	0	0	0	0
				2#	0	0	0	0	0
				3#	80	20	0	0	0
36	Male	54	III B	1#	70	30	20	0	0
				2#	90	0	0	0	0
				3#	90	60	0	0	0

### HLA-G Antibodies and Immunohistochemistry

Five anti-HLA-G murine antibodies were used in this study. mAbs 4H84 (dilution 1:200), MEM-G/1 (dilution 1:100) and MEM-G/2 (dilution 1:100), IgG1 antibodies detect denatured heavy chain of all HLA-G isoforms (Exbio, Prague, Czech Republic); mAbs 5A6G7 and 2A12, IgG1 antibodies probe denaturized heavy chain of HLA-G5/HLA-G6 isoforms (dilution 1:100; Exbio, Prague, Czech Republic). Immunohistochemistry assay was performed on 4-μm-thick, formalin-fixed and paraffin-embedded tumor lesion sections. Details of the protocols was according to our previous study (22). Immunohistochemistry staining was visualized with a Dako EnVison kit (Dako, Glostrup, Denmark). The percentage of HLA-G positive tumor cells was determined by presence of HLA-G staining while irrespective of staining intensity. HLA-G staining was evaluated by two reviewers who were blind to the patient clinicopathological information. Membrane or/and cytoplasmic expression of HLA-G were interpreted as positive. Percentage of HLA-G-positive tumor cells was determined by each observer, and the average of scores was calculated.

### Statistical Analysis

Statistical analysis was performed with the SPSS 13.0 statistical software package (SPSS, Inc., Chicago, IL, USA). Comparison between groups was analyzed with non-parametric Mann-Whitney U or Kruskal-Wallis H test. *p*<0.05 (two-tailed) was considered statistically significant.

## Results

**Figure 1:**
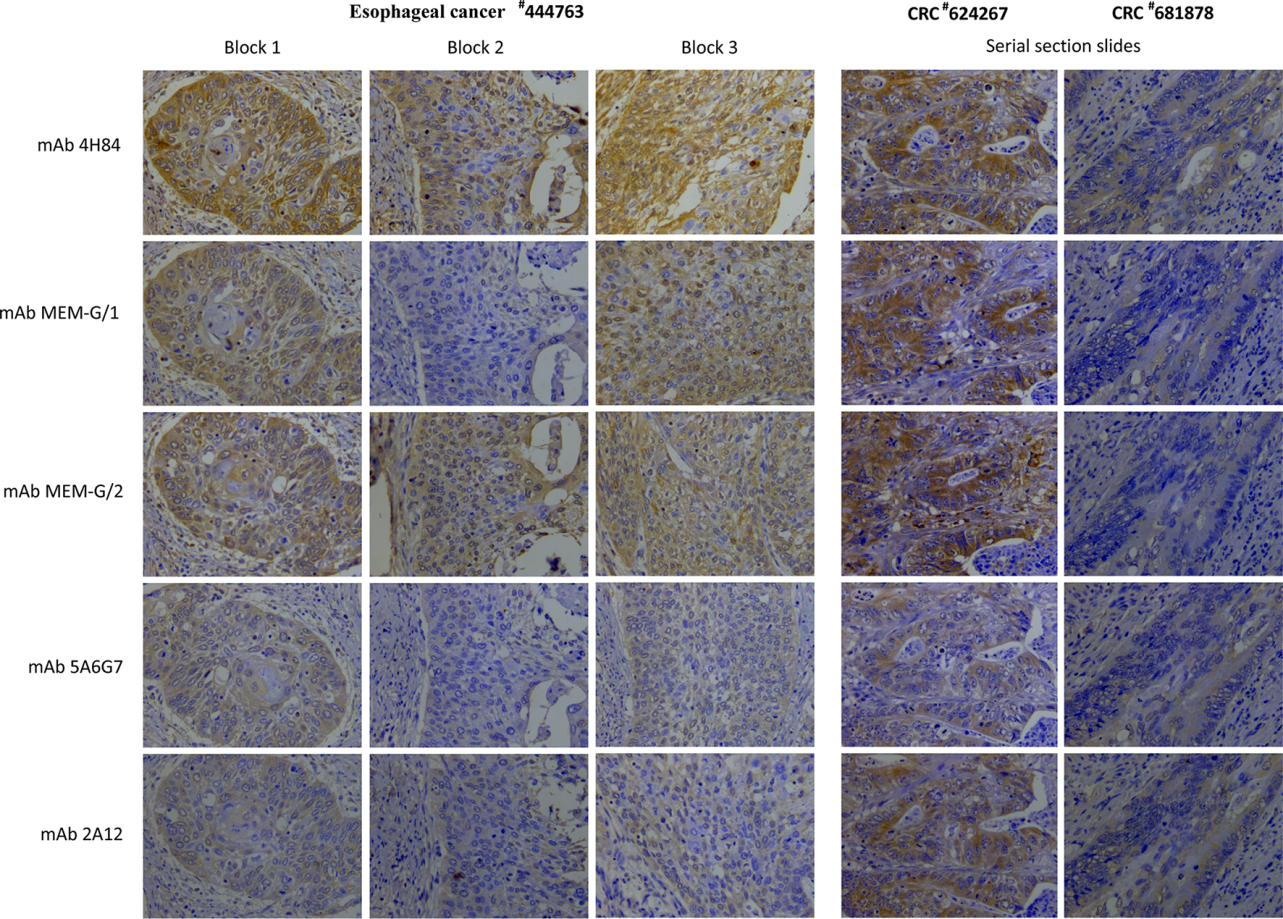
Representatives of intratumor heterogeneous staining of HLA-G expression with different anti-HLA-G antibodies in three blocks of a esophageal squamous cell carcinoma sample (^#^444763), and serial sections of two colorectal cancer samples (^#^624267 and ^#^681878) (400×).

To evaluate the heterogeneity of HLA-G expression in cancers, three different types of tumor tissue samples were prepared. a) For three CRC tissue samples (#598937, #624267 and #681878), 50 slides was serially sectioned for each sample. Among 50 slides, 10 randomized slides for each antibody probing. b) Slides from three different zones within a same sample from another five CRC samples (#1022488, #1022363, #1020932, #1023081, and #444345), and c) slides from three case-matched blocks that each includes 36 esophageal cancer samples. These slides were probed with five different anti-HLA-G antibodies. Anti-HLA-G antibodies were divided into two groups according to the specificity of these antibodies. Group 1: mAbs 4H84, MEM-G/1 and MEM-G/2, which detect denatured heavy chain of all HLA-G isoforms; Group 2: mAbs 5A6G7 and 2A12, which detect denaturized heavy chain of HLA-G5/HLA-G6 isoforms. The representative immunohistochemistry HLA-G staining patterns of CRC and ESCC were shown in **Figure 1**.

### Intratumor Heterogeneity of HLA-G

Intratumor heterogeneous expression of HLA-G was observed among different sections and antibodies used in three CRC tissue samples (#598937, #624267, and #681878).

For the Group 1 antibodies (mAbs 4H84, MEM-G/1, and MEM-G/2), HLA-G expression was dramatically different in samples CRC#598937 (*p*<0.001) and CRC#681878 (*p*<0.001), while comparable degree of HLA-G expression was observed in sample CRC#624267 (*p*=0.453). Among these samples, no significant variation of HLA-G expression was found for the Group 2 antibodies (mAbs 5A6G7 and 2A12; **Table 1**).

Moreover, HLA-G expression in samples from different zones of a same tumor also varied significantly when detected with a distinct anti-HLA-G antibody. CRC#1022488 for an example, the percentage of HLA-G expression detected with mAbs 5A6G7 and 2A12 are much higher than that probed with mAbs 4H84, MEM-G/1 and MEM-G/2. Zone 2 particularly, percentage of HLA-G expression detected by mAb 5A6G7 is 20.29% while HLA-G is nearly negative detected by mAb 4H84 (0.71%). In CRC#1022363, the degree of HLA-G detected by mAb 4H84 was 14.88%, 50.0% and 22.0% in zone 1, zone 2, and zone 3, respectively. HLA-G expression in zone 2 and 3 was almost undetectable, while HLA-G was positive in zone 1 when detected by mAbs MEM-G/1 and MEM-G/2. Moreover, HLA-G expression was observed in all three zones when detected with mAb 5A6G7 and mAb 2A12, respectively (**Table 2**). Similarly, intratumor heterogeneity of HLA-G expression was also found in case-matched esophageal cancer blocks (**Table 3**).

### Intratumor Heterogeneity of HLA-G Isoforms

**Figure 2:**
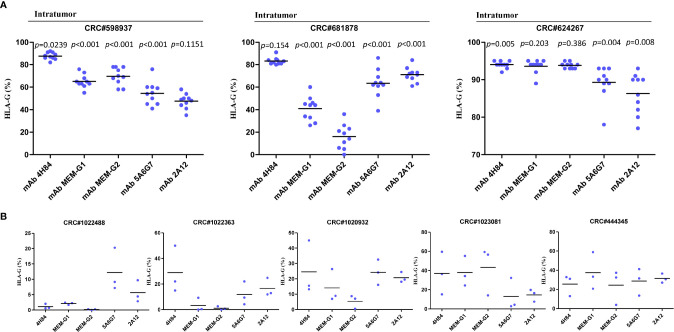
Intratumor heterogeneous staining of HLA-G expression with different anti-HLA-G antibodies in **(A)** serial sections of three colorectal cancer samples (each sample with 50 serial sections, 10 randomized slides for each antibody). Dot represents each section. Black line represents median. Comparison among the sections was analyzed with analyzed with Kruskal-Wallis H test. **(B)** three different zones within a same sample (five CRC samples). Dot represents each zone. Black line represents median.

Distinct pattern and variation of HLA-G expression was also observed for each antibody for HLA-G detection among 10 randomized slides from a same tumor sample. No significant variation of HLA-G expression was observed when detected by mAb 2A12 in CRC#598937 (*p*=0.1151), mAb 4H84 in CRC#681878 (*p*=0.154), and mAbs MEM-G/1 (*p*=0.203) and MEM-G/2 (*p*=0.386) in CRC#624267. HLA-G expression was found varied dramatically among 10 slides when probed with a distinct anti-HLA-G antibody (**Figure 2A**). To be noted, previously considered as unexpected immunohistochemistry staining patters such as mAb 4H84^neg^ mAb 5A6G7^pos^ was observed in this study (**Table 2**). In CRC#1022488, HLA-G expression is low/negative stained with mAbs 4H84, MEM-G/1, and MEM-G/2, while HLA-G is positive when stained with mAbs 5A6G7 and 2A12. This staining pattern now could be explained by the findings that novel HLA-G isoforms such as lacking the α1 domain was depicted by Tronik-Le Roux et al. (3) in a renal cancer study. Similar data were also observed in slides from three different zones within a same sample from another five CRC samples (#1022488, #1022363, #1020932, #1023081, and #444345; **Figure 2B**).

Among 36 ESCC samples, HLA-G expression could be detected by mAbs 4H84, MEM-G/1 and MEM-G/2, while HLA-G expression is negative detected by mAbs 5A6G7 and 2A12 in most cases. Moreover, the staining pattern for mAbs 4H84 and 5A6G7 seems more consistent according to their recognizing epitope in the HLA-G heavy chain, that no mAbs 4H84^neg^5A6G7^pos^ was observed (**Table 3**).

## Discussion

Inter-tumor HLA-G expression in various types of tumor tissues has been widely investigated and its clinical significance has been well acknowledged. A large body of studies have evidenced that higher degree of HLA-G expression in cancers is related to disease progression and worse clinical outcome (14). Based on the signaling pathway of HLA-G/ILTs and its clinical relevance, HLA-G as a potential immune checkpoints is expected (1). Though ICIs such as targeting the PD-L/PD-L1 is certainly an effective and promising strategic regime for cancer immunotherapy, limited effects of the ICIs therapy resulted from inter- and intratumor heterogeneous expression of immune checkpoints is gaining concern (19).

Indeed, the degree and percentage of HLA-G in cancers varies significantly among different types of cancers which have been observed to be negative in uveal melanoma to totally positive in hydatidiform moles (23, 24), and inconsistent HLA-G findings among different cohorts or laboratories existed in most cases even on a same type of cancer such as breast cancer (25–27) and CRC (22, 28–30). These controversies might be raised by the different specificities of HLA-G monoclonal antibodies, varied laboratory technical procedures, or different composition and HLA-G genetic backgrounds of the included cohorts (14, 31). In line with this, our data showed that different staining pattern of HLA-G expression has been observed between the CRC and ESCC, where HLA-G is almost negative in ESCC but positive in CRC samples when detected by mAbs 5A6G7 and 2A12. This finding indicated that HLA-G isoforms could be differentially regulated among different types of cancers. Moreover, mechanisms involved in regulation of HLA-G expression are complex. In addition to the HLA-G genetic variations both in 5′-upstream regulatory region and in 3′-untranslated region which comprise binding sites for transcription factor and microRNAs and epigenetic modifications (32), other environmental factor such as hypoxia, cytokines, hormones, and even immunotherapy chemicals and radiation have been acknowledged to be related to the regulation of HLA-G expression (33–35).

Intratumor heterogeneity of HLA-G expression has been firstly detailed in 19 primary renal cell cancer (RCC) tumor tissues. HLA-G expression was sharply differed either between samples or inside a tumor tissue (20). In that study, with mAb 4H84, authors revealed that various degree of HLA-G expression exists among different areas (zones) as they illustrated in sample RCC#2 (70% in area T1, 37% in T2, 58% in T3 and T4, respectively), while no HLA-G expression was observed in the T1 or T2 areas in sample RCC#10. In line with their findings, as our data in this study revealed that intratumor heterogeneous expression of HLA-G is a common phenomenon among different zones within a sample in CRC and ESCCs. According to these results, similar findings that intratumor HLA-G heterogeneity could be expected in other malignancies. Shortly afterwards, with transcriptome analysis in RCC samples, they further depicted that, besides the already identified HLA-G1~HLA-G7 isoforms, novel HLA-G isoforms without an α1 domain and transmembrane region could be existed (3). This important finding do explain previously unexpected immunohistochemistry staining patters such as mAb 4H84^neg^ mAb 5A6G7^pos^, which was observed in our study such as the CRC#1022488 and other samples. In this context, in an our previous study, we found 44 out of 379 (11.6%) CRC patients were with the staining pattern of mAbs 4H84^neg^ 5A6G7^pos^, and CRC patients with the patterns of mAbs 4H84^neg^ 5A6G7^pos^ had a longer survival time than those with the pattern of mAbs 4H84^pos^5A6G7^neg^ (36). However, future investigations for the biological functions and clinical significance of novel HLA-G isoforms with mAbs 4H84^neg^ 5A6G7^pos^ are extremely necessary.

However, our study have notable limitations. First, this study is based on a very limited size of patients and types of cancers, the real-world of the heterogeneity of HLA-G expression in more different types of cancers and in larger cohorts of cancer patients remain to be explored. Second, being the very limited size of the patients included, clinical significance of the heterogeneity of HLA-G and HLA-G isoform expression in cancers is still unknown. Third, potential mechanisms underlying the heterogeneity of HLA-G in cancers remain to be uncovered. Finally, more specific antibodies for HLA-G isoforms are needed to define the clinical significance of a particular HLA-G isoforms.

In summary, our study revealed a rather high degree of intratumor heterogeneity of HLA-G expression in cancers, and degree of HLA-G expression is also varied among anti-HLA-G antibodies with different specificities. Therefore, to evaluate the clinical significance of HLA-G expression in cancers, important issues including location of the tumor tissues isolated, HLA-G isoforms and specificity of the anti-HLA-G antibodies should be concerned.

## Data Availability Statement

The original contributions presented in the study are included in the article. Further inquiries can be directed to the corresponding author.

## Ethics Statement

The studies involving human participants were reviewed and approved by Medical Ethics Review Board of Taizhou Hospital of Zhejiang Province. The patients/participants provided their written informed consent to participate in this study.

## Author Contributions

W-HY: study design. XZ, JG, and AL: performed experiments. J-GZ, Q-YH, Q-YC, Y-HY, W-JZ, and H-HX: material support and data acquisition. W-HY: performed statistical analysis and drafted the manuscript. All authors contributed to the article and approved the submitted version.

## Funding

This work was supported by grants from National Natural Science Foundation of China (81901625) and Science and Technology Bureau of Taizhou (1901ky01; 1901ky04, 1901ky05, 1901ky09).

## Conflict of Interest

The authors declare that the research was conducted in the absence of any commercial or financial relationships that could be construed as a potential conflict of interest.

## References

[1] Carosella ED Rouas-Freiss N Tronik-Le Roux D Moreau P LeMaoult J HLA-G: An Immune Checkpoint Molecule. Adv Immunol (2015) 127:33–144. 10.1016/bs.ai.2015.04.001 26073983

[2] Amiot L Vu N Samson M Biology of the immunomodulatory molecule HLA-G in human liver diseases. J Hepatol (2015) 62:1430–7. 10.1016/j.jhep.2015.03.007 25772038

[3] Tronik-Le Roux D Renard J Vérine J Renault V Tubacher E LeMaoult J Novel landscape of HLA-G isoforms expressed in clear cell renal cell carcinoma patients. Mol Oncol (2017) 11:1561–78. 10.1002/1878-0261.12119 PMC5664004 28815885

[4] Carosella ED Ploussard G LeMaoult J Desgrandchamps FA Systematic Review of Immunotherapy in Urologic Cancer: Evolving Roles for Targeting of CTLA-4, PD-1/PD-L1, and HLA-G. Eur Urol (2015) 68:267–79. 10.1016/j.eururo.2015.02.032 25824720

[5] Paul P Rouas-Freiss N Khalil-Daher I Moreau P Riteau B Le Gal FA HLA-G expression in melanoma: a way for tumor cells to escape from immunosurveillance. Proc Natl Acad Sci U S A (1998) 95:4510–5. 10.1073/pnas.95.8.4510 PMC22520 9539768

[6] Chen BG Xu DP Lin A Yan WH NK cytolysis is dependent on the proportion of HLA-G expression. Hum Immunol (2013) 74:286–9. 10.1016/j.humimm.2012.12.005 23238216

[7] Lin A Zhang X Xu HH Xu DP Ruan YY Yan WH HLA-G expression is associated with metastasis and poor survival in the Balb/c nu/nu murine tumor model with ovarian cancer. Int J Cancer (2012) 131:150–7. 10.1002/ijc.26375 21858813

[8] Loumagne L Baudhuin J Favier B Montespan F Carosella ED Rouas-Freiss N In vivo evidence that secretion of HLA-G by immunogenic tumor cells allows their evasion from immunosurveillance. Int J Cancer (2014) 135:2107–17. 10.1002/ijc.28845 24623585

[9] Lesport E Baudhuin J Sousa S LeMaoult J Zamborlini A Rouas-Freiss N Inhibition of human gamma delta [corrected] T-cell antitumoral activity through HLA-G: implications for immunotherapy of cancer. Cell Mol Life Sci (2011) 68:3385–99. 10.1007/s00018-011-0632-7 PMC11114898 21337044

[10] Naji A Menier C Morandi F Agaugué S Maki G Ferretti E Binding of HLA-G to ITIM-bearing Ig-like transcript 2 receptor suppresses B cell responses. J Immunol (2014) 192:1536–46. 10.4049/jimmunol.1300438 24453251

[11] Liang S Ristich V Arase H Dausset J Carosella ED Horuzsko A Modulation of dendritic cell differentiation by HLA-G and ILT4 requires the IL-6–STAT3 signaling pathway. Proc Natl Acad Sci U S A (2008) 105:8357–62. 10.1073/pnas.0803341105 PMC2448841 18550825

[12] Lee CL Guo Y So KH Vijayan M Guo Y Wong VH Soluble human leukocyte antigen G5 polarizes differentiation of macrophages toward a decidual macrophage-like phenotype. Hum Reprod (2015) 30:2263–74. 10.1093/humrep/dev196 26307092

[13] Köstlin N Ostermeir AL Spring B Schwarz J Marmé A Walter CB HLA-G promotes myeloid-derived suppressor cell accumulation and suppressive activity during human pregnancy through engagement of the receptor ILT4. Eur J Immunol (2017) 47:374–84. 10.1002/eji.201646564 27859042

[14] Lin A Yan WH Heterogeneity of HLA-G Expression in Cancers: Facing the Challenges. Front Immunol (2018) 9:2164. 10.3389/fimmu.2018.02164 30319626 PMC6170620

[15] Lin A Yan WH Intercellular transfer of HLA-G: its potential in cancer immunology. Clin Transl Immunol (2019) 8:e1077. 10.1002/cti2.1077 PMC6716982 31489189

[16] Agaugué S Carosella ED Rouas-Freiss N Role of HLA-G in tumor escape through expansion of myeloid-derived suppressor cells and cytokinic balance in favor of Th2 versus Th1/Th17. Blood (2011) 117:7021–31. 10.1182/blood-2010-07-294389 21482709

[17] Lin A Xu HH Xu DP Zhang X Wang Q Yan WH Multiple steps of HLA-G in ovarian carcinoma metastasis: alter NK cytotoxicity and induce matrix metalloproteinase-15 (MMP-15) expression. Hum Immunol (2013) 74:439–46. 10.1016/j.humimm.2012.11.021 23228395

[18] Wang C Chen J Zhang Q Li W Zhang S Xu Y Elimination of CD4(low)HLA-G(+) T cells overcomes castration-resistance in prostate cancer therapy. Cell Res (2018) 28:1103–17. 10.1038/s41422-018-0089-4 PMC6218442 30297869

[19] Jiang Y Zhao X Fu J Wang H Progress and Challenges in Precise Treatment of Tumors With PD-1/PD-L1 Blockade. Front Immunol (2020) 11:339. 10.3389/fimmu.2020.00339 32226426 PMC7080697

[20] Rouas-Freiss N LeMaoult J Verine J Tronik-Le Roux D Culine S Hennequin C Intratumor heterogeneity of immune checkpoints in primary renal cell cancer: Focus on HLA-G/ILT2/ILT4. Oncoimmunology (2017) 6:e1342023. 10.1080/2162402X.2017.1342023 28932645 PMC5599087

[21] Edge SB Compton CC The American Joint Committee on Cancer: the 7th edition of the AJCC cancer staging manual and the future of TNM. Ann Surg Oncol (2010) 17:1471–4. 10.1245/s10434-010-0985-4 20180029

[22] Zhang RL Zhang X Dong SS Hu B Han QY Zhang JG Predictive value of different proportion of lesion HLA-G expression in colorectal cancer. Oncotarget (2017) 8:107441–51. 10.18632/oncotarget.22487 PMC5746078 29296176

[23] Anastassiou G Rebmann V Wagner S Bornfeld N Grosse-Wilde H Expression of classic and nonclassic HLA class I antigens in uveal melanoma. Invest Ophthalmol Vis Sci (2003) 44:2016–9. 10.1167/iovs.02-0810 12714638

[24] Goldman-Wohl D Ariel I Greenfield C Hochner-Celnikier D Lavy Y Yagel S A study of human leukocyte antigen G expression in hydatidiform moles. Am J Obstet Gynecol (2001) 185:476–80. 10.1067/mob.2001.115994 11518912

[25] Chen HX Lin A Shen CJ Zhen R Chen BG Zhang X Upregulation of human leukocyte antigen-G expression and its clinical significance in ductal breast cancer. Hum Immunol (2010) 71:892–8. 10.1016/j.humimm.2010.06.009 20547193

[26] He X Dong DD Yie SM Yang H Cao M Ye SR HLA-G expression in human breast cancer: implications for diagnosis and prognosis, and effect on allocytotoxic lymphocyte response after hormone treatment in vitro. Ann Surg Oncol (2010) 17:1459–69. 10.1245/s10434-009-0891-9 20052552

[27] da Silva GB Silva TG Duarte RA Neto NL Carrara HH Donadi EA Expression of the Classical and Nonclassical HLA Molecules in Breast Cancer. Int J Breast Cancer (2013) 2013:250435. 10.1155/2013/250435 24363939 PMC3864140

[28] Swets M König MH Zaalberg A Dekker-Ensink NG Gelderblom H van de Velde CJ HLA-G and classical HLA class I expression in primary colorectal cancer and associated liver metastases. Hum Immunol (2016) 77:773–9. 10.1016/j.humimm.2016.03.001 26968946

[29] Ye SR Yang H Li K Dong DD Lin XM Yie SM Human leukocyte antigen G expression: as a significant prognostic indicator for patients with colorectal cancer. Mod Pathol (2007) 20:375–83. 10.1038/modpathol.3800751 17277760

[30] Guo ZY Lv YG Wang L Shi SJ Yang F Zheng GX Predictive value of HLA-G and HLA-E in the prognosis of colorectal cancer patients. Cell Immunol (2015) 293:10–6. 10.1016/j.cellimm.2014.10.003 25461612

[31] Swets M Wouters A Krijgsman D van Vlierberghe RLP Boot A van Eendenburg JD HLA-G protein expression in colorectal cancer evaluated by immunohistochemistry and western blot analysis: Its expression characteristics remain enigmatic. Clin Immunol (2018) 194:80–6. 10.1016/j.clim.2018.07.005 30006120

[32] Castelli EC Veiga-Castelli LC Yaghi L Moreau P Donadi EA Transcriptional and posttranscriptional regulations of the HLA-G gene. J Immunol Res (2014) 2014:734068. 10.1155/2014/734068 PMC3987962 24741620

[33] Ziliotto M Rodrigues RM Chies JAB Controlled hypobaric hypoxia increases immunological tolerance by modifying HLA-G expression, a potential therapy to inflammatory diseases. Med Hypotheses (2020) 140:109664. 10.1016/j.mehy.2020.109664 32155542

[34] Persson G Bork JBS Isgaard C Larsen TG Bordoy AM Bengtsson MS Cytokine stimulation of the choriocarcinoma cell line JEG-3 leads to alterations in the HLA-G expression profile. Cell Immunol (2020) 352:104110. 10.1016/j.cellimm.2020.104110 32387976

[35] Ishikawa M Brooks AJ Fernández-Rojo MA Medina J Chhabra Y Minami S Growth hormone stops excessive inflammation after partial hepatectomy allowing liver regeneration and survival via induction of H2-Bl/HLA-G. Hepatology (2020). 10.1002/hep.31297 PMC7894545 32342533

[36] Lin A Zhang X Zhang RL Zhang JG Zhou WJ Yan WH Clinical Significance of Potential Unidentified HLA-G Isoforms Without α1 Domain but Containing Intron 4 in Colorectal Cancer Patients. Front Oncol (2018) 8:361. 10.3389/fonc.2018.00361 30234020 PMC6131604

